# Monument to Dr. Horace Wells, the Discoverer of Anaesthesia

**Published:** 1870-05

**Authors:** 


*Monument to Dr. Horace Wells, the Discoverer of Anæsthesia.*—The Dental Association of Hartford, Connecticut, are about to erect in that city a suitable monument or statue to the memory of Dr. Horace Wells, formerly a practicing Dentist, in honor of the great discovery which has conferred such blessings on suffering humanity. We are pleased to learn of this interest on the part of the Dental Association, for the proof is so conclusive that to Dr. Wells belongs the honor of the discovery, that no honorable and unbiased person can ignore it. Prof. J. Y. Simpson, of the University of Edinburg, Scotland, in a late paper, extracts from which we published in the last No. of the Journal, awards to Dr. Wells the merit of "extracting teeth from a dozen or more patients, rendered insensible by inhaling nitrous oxide gas" at least two years previous to the time when Drs. Jackson and Morton resorted to its use.

At a regular meeting of the Hartford Society of Dentists, held at the office of Dr C. M. Hooker, April 11, 1870. the following resolutions, presented by Dr. McManus, were adopted:—

*Resolved,* That the natural gratitude due to the memory of public benefactors, imperatively demands that the city of Hartford and the State of Connecticut, with the medical and dental professions, cause a suitable monument to be erected in the public park of this city, in memory of Dr. Horace Wells, the discoverer of anæsthesia.

*Resolved,* That Drs. J. M. Riggs. James McManus, E. E. Crofoot, C. M. Hooker and Wm. Blatchley, be and are hereby appointed a committee, whose duty it shall be to cause suitable petitions to be presented to the city council of Hartford and the general Assembly of this State in furtherance of this object, and to obtain signatures to the same.

